# Supplementary material for: Inbreeding depression in one of the last DFTD-free wild populations of Tasmanian devils
Source: PeerJ. 2020 Jun 16;8:e9220. doi: 10.7717/peerj.9220 (PMC7304431; doi:10.7717/peerj.9220)
Supplement: Supplemental Information 1 — - Table S1: Full model sets corresponding to final models provided in Table 2, examining predictors of reproductive success in female Tasmanian devils. - Table S2: Predictors of litter size in female Tasmanian devils, when accounting for year as an additional random effect. Predictors have been standardised, and information theory used to identify the best model (model averaging not required; full model set provided in Table S3). - Table S3: Full model set corresponding to the final model provided in Table S2 - Supplementary Results: Alternative modelling of devil reproductive success. [file peerj-08-9220-s001.docx]

**Supplementary material to: Gooley et al. “Inbreeding depression in one of the last DFTD-free wild populations of Tasmanian devils”, submitted to PeerJ.**

**Supplementary Table S1:** Full model sets corresponding to final models provided in Table 2, examining predictors of reproductive success in female Tasmanian devils.

| **Model** | $\boldsymbol{\beta}_{\boldsymbol{0}}$ | $\boldsymbol{\beta}_{\boldsymbol{age}}$ | $\boldsymbol{\beta}_{\boldsymbol{IR}}$ | $\boldsymbol{\beta}_{\boldsymbol{year}}$ | **DF** | **logLik** | **AIC_C_** | **ΔAIC_C_** | ***w_i_*** |
| --- | --- | --- | --- | --- | --- | --- | --- | --- | --- |
| Litter size | -1.070 |  | -0.955 | -2.333 | 3 | -137.4 | 281.0 | 0.00 | 0.640 |
|  | -1.077 | 0.296 | -0.948 | -2.428 | 4 | -136.8 | 282.2 | 1.17 | 0.356 |
|  | -1.033 |  |  | -2.280 | 2 | -143.9 | 292.0 | 11.03 | 0.003 |
|  | -1.041 | 0.309 |  | -2.378 | 3 | -143.3 | 293.0 | 11.96 | 0.002 |
|  | -0.873 | -0.524 | -0.907 |  | 3 | -179.7 | 365.7 | 84.74 | 0.000 |
|  | -0.857 |  | -0.869 |  | 2 | -182.1 | 368.3 | 87.28 | 0.000 |
|  | -0.832 | -0.477 |  |  | 2 | -186.6 | 377.4 | 96.38 | 0.000 |
|  | -0.821 |  |  |  | 1 | -188.6 | 379.3 | 98.35 | 0.000 |
| Breeding | -0.480 |  |  | -2.427 | 2 | -47.3 | 98.6 | 0.00 | 0.309 |
|  | -0.487 |  | -0.736 | -2.477 | 3 | -46.2 | 98.7 | 0.01 | 0.306 |
|  | -0.494 | 0.599 |  | -2.620 | 3 | -46.6 | 99.5 | 0.90 | 0.197 |
|  | -0.502 | 0.612 | -0.737 | -2.676 | 4 | -45.6 | 99.6 | 0.99 | 0.188 |
|  | -0.405 |  |  |  | 1 | -60.6 | 123.2 | 24.54 | 0.000 |
|  | -0.416 |  | -0.635 |  | 2 | -59.5 | 123.2 | 24.57 | 0.000 |
|  | -0.407 | -0.260 |  |  | 2 | -60.4 | 124.9 | 26.28 | 0.000 |
|  | -0.419 | -0.279 | -0.647 |  | 3 | -59.3 | 125.0 | 26.31 | 0.000 |
| Litter size 1+ | 1.328 | -0.843 | -1.073 | -0.720 | 4 | -38.9 | 87.2 | 0.00 | 0.340 |
|  | 1.292 | -0.962 | -1.072 |  | 3 | -40.4 | 87.6 | 0.43 | 0.274 |
|  | 1.280 |  | -0.959 | -0.854 | 3 | -40.8 | 88.4 | 1.24 | 0.182 |
|  | 1.228 |  | -0.932 |  | 2 | -43.2 | 90.7 | 3.50 | 0.059 |
|  | 1.248 | -0.669 |  | -0.726 | 3 | -42.1 | 90.9 | 3.75 | 0.052 |
|  | 1.218 |  |  | -0.836 | 2 | -43.5 | 91.3 | 4.10 | 0.044 |
|  | 1.212 | -0.791 |  |  | 2 | -43.7 | 91.8 | 4.58 | 0.034 |
|  | 1.174 |  |  |  | 1 | -45.8 | 93.7 | 6.47 | 0.013 |

Abbreviations: DF = degrees of freedom, logLik = log likelihood, AIC_C_ = Akaike information criterion corrected for sample size, ΔAIC_C_ = change in AIC_C_ relative to the top-ranked model in the set, *w_i_* = Akaike weight

**Supplementary Results**

We attempted to run our models using year as both a random and fixed factor using the package lme4 (Bates & Maechler 2009), e.g.

$$logit\left( \frac{litter size}{4} \right)=\beta_{0}+\beta_{1}{IR}_{i}+\beta_{2}{age}_{i}+\beta_{3}{year}_{i}+(1|{year}_{i})+\varepsilon_{i}$$

Unfortunately only the model for our first response (litter size) converged. The other two responses (breeding, and litter size 1+) failed, even when the fixed effects were simplified (e.g. by removing “age”). Nevertheless, comparing these litter size results (Supplementary Table S2) with the corresponding results of our main analysis (Table 2), it can be seen that the two sets of results are qualitatively similar. The two predictors with substantial effects (IR and Year) were important in both models, with effect sizes in the same direction and of similar magnitude across models. Age did not appear in the revised model (Table S2), but was poorly supported also in the main analysis (Table 2). In addition, the *R^2^* estimates are of a similar magnitude for both model structures.

**Supplementary Table S2:** Predictors of litter size in female Tasmanian devils, when accounting for year as an additional random effect. Predictors have been standardised, and information theory used to identify the best model (model averaging not required; full model set provided in Supplementary Table S3).

| **Model** | **N** | **Predictor** | **Estimate** | **SE** | **RI^*^** | **Marginal *R^2^^*** | **Conditional *R*^2^^** |
| --- | --- | --- | --- | --- | --- | --- | --- |
| Litter size | 90 | Intercept | -1.059 | 0.242 |  | 0.363 | 0.397 |
|  |  | Age | - | - | 0 |  |  |
|  |  | IR | -1.042 | 0.283 | 1.00 |  |  |
|  |  | Year | -2.567 | 0.480 | 1.00 |  |  |

Abbreviations: N = sample size, SE = standard error, IR = internal relatedness, RI = relative importance (sum of Akaike weights)

^*^ Note, only one top model identified, model averaging not required

^^^ Marginal and conditional R^2^ evaluated using the R-package performance (Lüdecke *et al.* 2017) using the method of Nakagawa *et al.* (2017)

**Supplementary Table S3:** Full model set corresponding to the final model provided in Supplementary Table S2

| **Model** | $\boldsymbol{\beta}_{\boldsymbol{0}}$ | $\boldsymbol{\beta}_{\boldsymbol{age}}$ | $\boldsymbol{\beta}_{\boldsymbol{IR}}$ | $\boldsymbol{\beta}_{\boldsymbol{year}}$ | **DF** | **logLik** | **AIC_C_** | **ΔAIC_C_** | ***w_i_*** |
| --- | --- | --- | --- | --- | --- | --- | --- | --- | --- |
| Litter size | -1.058 |  | -1.042 | -2.575 | 4 | -135.2 | 278.8 | 0.00 | 0.744 |
|  | -1.058 | 0.062 | -1.037 | -2.585 | 5 | -135.2 | 281.0 | 2.21 | 0.246 |
|  | -0.518 |  | -1.067 |  | 3 | -141.1 | 288.5 | 9.67 | 0.006 |
|  | -0.531 | -0.131 | -1.079 |  | 4 | -141.0 | 290.6 | 11.73 | 0.002 |
|  | -1.010 |  |  | -2.475 | 3 | -142.5 | 291.2 | 12.42 | 0.001 |
|  | -1.011 | 0.163 |  | -2.506 | 4 | -142.4 | 293.2 | 14.36 | 0.001 |
|  | -0.456 |  |  |  | 2 | -148.6 | 301.3 | 22.50 | 0.000 |
|  | -0.454 | 0.021 |  |  | 3 | -148.6 | 303.5 | 24.64 | 0.000 |

**Literature cited in Supplementary Material**

Bates D, Maechler M (2009) lme4: Linear mixed-effects models using S4 classes. Available at <http://CRAN.R-project.org/package=lme4>.

Lüdecke D, Makowski D, Waggoner P, Patil I (2017) performance: Assessment of regression models performance. Available at: <https://cloud.r-project.org/web/packages/performance/index.html>.

Nakagawa S, Johnson PCD, Schielzeth H (2017) The coefficient of determination *R*^2^ and intra-class correlation coefficient from generalized linear mixed-effects models revisited and expanded. *Journal of The Royal Society Interface*, **14**, 20170213.
